# Significance of echocardiographic metrics including TRV and TAPSE/SPAP in mild haemodynamic pulmonary hypertension – data from EVIDENCE-PAH UK

**DOI:** 10.1186/s44156-026-00119-1

**Published:** 2026-06-08

**Authors:** Nina Karia, Daniel Murphy, Luke Howard, Martin Johnson, David G. Kiely, James Lordan, Colm McCabe, Joanna Pepke-Zaba, Daniel Knight, Vivek Muthurangu, J. Gerry Coghlan

**Affiliations:** 1 National Pulmonary Hypertension Service, Royal Free Hospital London NHS Foundation Trust, Pond Street, London, NW3 2QG UK; 2 Institute of Cardiovascular Science, University College London, London, WC1E 6BT UK; 3 National Pulmonary Hypertension Service, Hammersmith Hospital, London, W12 0HS UK; 4 Scottish Pulmonary Vascular Unit, Golden Jubilee National Hospital, Glasgow, G81 4DY UK; 5 Sheffield Pulmonary Vascular Disease Unit, Royal Hallamshire Hospital, Sheffield Teaching Hospitals NHS Foundation Trust, Sheffield, S10 2JF UK; 6 Pulmonary Vascular Unit, Freeman Hospital, Newcastle upon Tyne, NE7 7DN UK; 7 National Pulmonary Hypertension Service, Royal Brompton Hospital, London, SW3 6NP UK; 8 National Heart and Lung Institute, Imperial College, London, SW3 6LY UK; 9 Pulmonary Vascular Disease Unit, Royal Papworth Hospital NHS Foundation Trust, Cambridge, CB2 0AY UK

**Keywords:** Pulmonary hypertension, Mild elevations of pulmonary artery pressure, Right heart, Tricuspid regurgitation velocity (TRV), Tricuspid annular plane systolic excursion (TAPSE), Prognosis

## Abstract

**Background:**

The 2022 ESC/ERS guidelines redefined pulmonary hypertension (PH) as a mean pulmonary artery pressure (mPAP) >20 mmHg on right heart catheterisation (RHC). Echocardiography, using metrics such as tricuspid regurgitation velocity (TRV), tricuspid annular plane systolic excursion (TAPSE), and TAPSE/systolic pulmonary artery pressure (TAPSE/SPAP), guides referral for RHC. However, the few studies evaluating echocardiographic performance using the ESC/ERS 2022 thresholds have combined the newly included population as a relatively modest subgroup within their overall analysis.

**Results:**

We present a sample of 1,991 individuals from the EVIDENCE-PAH UK database. Our data demonstrate higher TRV and sPAP values and lower TAPSE/SPAP values with higher haemodynamic category. ROC analysis demonstrates that TRV, SPAP and TAPSE/SPAP perform well as predictors of mPAP > 20 mmHg and ≥25 mmHg (AUCs 0.785–0.830), but less well at predictive mPAP 21–24 mmHg (AUCs 0.656–0.686). In all PH patients and the mild PH (21–24 mmHg) subgroup, TAPSE/SPAP < 0.31 mm/mmHg predicted reduced survival (HR 2.01–3.14, *p* < 0.001). Similarly, in haemodynamically mild PH, TRV > 3.4 m/s was associated with worse survival compared to TRV < 2.5 m/s or 2.5–2.8 m/s (*p* < 0.001).

**Conclusions:**

While established echocardiographic metrics (TRV, TAPSE/SPAP) are strong predictors of significant PH (mPAP ≥25 mmHg), they are less accurate for mild PH (mPAP 21–24 mmHg). Importantly, mild PH itself is associated with increased mortality, and within this group, TRV >3.4 m/s and TAPSE/SPAP <0.31 mm/mmHg identify patients at highest risk, supporting their prognostic utility even in early haemodynamic disease.

**Supplementary information:**

The online version contains supplementary material available at 10.1186/s44156-026-00119-1.

## Introduction

In 2022, the ESC/ERS guidelines lowered the haemodynamic threshold for the diagnosis of pulmonary hypertension (PH) to a mean pulmonary artery pressure (mPAP) >20 mmHg [1] from the historical threshold of ≥25 mmHg. The update is underpinned by evidence showing that mild elevation in pulmonary artery pressures is associated with poorer outcomes [2]. Despite this lowering of thresholds, the echo thresholds assessing probability of PH have remained the same, in order to avoid an increased false positive rate that could lead to unnecessary invasive haemodynamic tests and its associated risks.

While the gold standard for diagnosing PH is right heart catheterisation (RHC), echocardiography is often the initial investigation that raises suspicion of PH and therefor plays a significant role in evaluating patients with *suspected* disease. Single-measure echocardiographic metrics, such as tricuspid regurgitation velocity (TRV) and tricuspid annular plane systolic excursion (TAPSE) are routinely used in the assessment of PH [1, 3–5], and elevated TRV values are highly predictive of PH [6].

Currently, ESC/ERS guidelines use TRV to stratify patients into low (TRV < 2.8 m/s), intermediate (2.9–3.4 m/s) and high (>3.4 m/s) probability of PH [1, 3, 4]. These cut-offs have been adopted in national echocardiography protocols [7, 8]. However, caution is required with this method, as the absence of tricuspid regurgitation does not exclude a diagnosis of PH. In recognition of this limitation, the ESC/ERS guidelines recommend that additional echocardiographic features are considered, with right atrial, right ventricular and pulmonary arterial parameters also requiring consideration before attributing a probability of PH if the TRV is <3.4 m/s. Finally, it is worth noting that lower thresholds of TRV ≥ 2.5 m/s have also been associated with a worse outcome [9].

The TRV can also be converted tricuspid regurgitation gradient (TRG) by using the simplified Bernoulli’s equation: TRG = 4(TRV^2^). This correlates to systolic pulmonary artery pressure (SPAP) from invasive haemodynamics by RHC, although this correlation has been shown to lack precision [1, 10]. In reality, although TRV is the recommended measure in guidelines, it is often the estimated pulmonary artery systolic pressure (ePASP) that is documented in summary reports which triggers a clinician to refer. ePASP is calculated using the Eq. 4(TRV^2^) + right atrial pressure (RAP). This can lead to further potential amplification in errors [8], as studies have shown interpretation of RAP estimation by echocardiography frequently inaccurate [1, 11, 12]. However, there has been a drive to standardise this in more recent echocardiography guidelines [7, 8] leading to greater consistency in echocardiographic reporting and practice.

Recently, combined metrics such as TAPSE/SPAP have been demonstrated to provide additional diagnostic and prognostic information in individuals with PH. TAPSE/SPAP has been proposed as a useful echocardiographic surrogate for right ventricle–pulmonary artery (RV–PA) coupling, showing good correlation with Ees/Ea measurements obtained by RHC [13]. TAPSE/SPAP has also been shown to correlate with haemodynamic and functional metrics in PH, as well as predicting mortality [14, 15]. Furthermore, TRV/TAPSE has been shown to correlate with New York Heart Association (NYHA) functional class and SPAP measurement by RHC [16].

The diagnostic and prognostic capabilities of echocardiography make it the first line tool in PH. Although, these metrics have been validated in a PH population, the majority of studies examining the role of TAPSE/SPAP have been conducted prior to the adoption of the 2022 guidelines, which redefined PH as a mPAP > 20 mmHg. This change introduced a new cohort of individuals with mPAP 21–24 mmHg, in whom these metrics have not been studied as thoroughly. There have been some studies that have attempted to validate echocardiographic metrics in PH patients defined using the revised mPAP > 20 mmHg threshold. However, individuals with a mPAP 21–24 mmHg were underrepresented, skewing the results towards those with more severe haemodynamics.

EVIDENCE-PAH UK [17] addresses this limitation. As a large UK based cohort, the dataset is enriched with patients who have mild elevations of haemodynamics (mPAP 21–24 mmHg), enabling more detailed analysis of this understudied group [17]. We use our EVIDENCE-PAH UK population to evaluate the diagnostic and prognostic utility of routinely used echocardiographic parameters; TRV, sPAP, TAPSE and TAPSE/sPAP, in our mild elevation of haemodynamic population.

## Methods

### EVIDENCE-PAH UK population [17]

EVIDENCE-PAH UK is a national haemodynamic study focussing on mild elevations of pulmonary artery pressure. Haemodynamic data was prospectively collected as part of the UK National Pulmonary hypertension audit from all UK tertiary pulmonary hypertension centres. Patients were stratified by haemodynamics into three groups; mPAP < 21 mmHg, 21–24 mmHg and ≥25 mmHg. The full study protocol has been published previously [17]. Multiple other clinical and investigation parameters were collected, including echocardiographic metrics. Echocardiography parameters were collected through review of local reports collected retrospectively where data was missing.

### ECHO parameters

Of the EVIDENCE PAH UK population, we sampled all patients who had a TRV available. We choose this parameter as it was reliably available and recommended to stratify probability of PH by echocardiography in previous and redefined ESC/ERS guidelines [1, 3, 4]. Full echocardiographic parameters recorded in EVIDENCE PAH can be found in the supplement (Supplementary Table 1).

Right atrial pressure (RAP) was recalculated to standardise changes in guidelines over time. By current guidance, RAP was derived by IVC dimensions (normal ≤ 2.1 cm, dilated > 2.1 cm) and response to inspiration (normal ≥ 50%), stratifying into three group (3 mmHg, 8 mmHg and 15 mmHg) based on IVC metrics [7, 8].

Calculation of ePASP was based on Bernoulli’s equation: 4(TRV^2^) + RAP. Echocardiographic assessment of RAP data by echocardiography based on IVC measurements and collapsibility where used. Where RAP data was not available, RAP as measured at the time of RHC was used to keep measurements as close to what is clinically available; this was felt to be a reasonable adjustment, this data is available in supplementary table 1.

For TAPSE/SPAP, diagnostic and prognostic thresholds of 0.55 mm/mmHg and 0.31 mm/mmHg, respectively, were used [1, 13].

Although we prioritised quantitative data, where this was not available qualitative and visual data was used. Where possible volumetric data was prioritised over area, diameter and then visual estimates according to the priorities in Supplementary Table 2, this table also demonstrates the number of patients who had quantitative measurements available for additional echo metrics.

### Statistical methods

Records from our database without TRV values were excluded from analysis. For categorical variables including presence or absence of RA dilatation, LA dilatation, LVH, reduced LVEF, LV diastolic dysfunction, and mitral regurgitation, continuous measurements (e.g. RA area or LV wall thickness) were used where available in accordance with British Society of Echocardiography guidance (see Supplementary Table 2). Where not available, the visual impression of the reporting echocardiographer was used. Summary statistics were calculated using mean and standard deviation for normally-distributed variables and median and Q1–Q3 range for others. Across groups, continuous variables were compared by Fisher’s ANOVA if variances were approximately equal and by Welch’s ANOVA if not. Homoscedasticity was assessed visually using Q–Q plots. Where post-hoc testing was required, Tukey’s test was utilised for Fisher’s ANOVA and the Games–Howell test was utilised for Welch’s ANOVA. Categorical variables were compared across groups using the chi-squared test, applying the Bonferroni correction for multiple comparisons. Diagnostic threshold testing was performed using receiver operating characteristic (ROC) analysis with the reporting of aurea-under-curve (AUC) values. For biomarkers for which a lower value is hypothesised to result in a higher likelihood of diagnosis, such as TAPSE/SPAP, an inverse test (i.e. testing for the normal condition rather than the disease condition) was performed. For testing differences in survival between groups, the log-rank test was utilised, applying the Holm–Bonferroni correction for multiple comparisons for pair-wise testing. Individual Cox regression models were constructed for variables of interest. Age and individual sex were used as covariates for each model for adjustment. A multivariate Cox regression model for survival was constructed with age, sex, TRV, and TAPSE as covariates. TRV and sPAP were found to have significant positive collinearity and so sPAP was excluded as a covariate. Kaplan-Meier plots were utilised to demonstrate survival at prognostic thresholds. Analysis and graphing were performed using R v4.4.2 (R Foundation for Statistical Computing) and RStudio v2024.12.0 (RStudio, PBC). Receiver operating characteristic (ROC) analysis was performed using the *plotROC* R package. Survival analysis was performed using the *survival* R package.

### Ethics

Ethical approval is under the EVIDENCE-PAH UK study, granted by the national ethics committee, Health Research Authority, London-Harrow Research committee REC reference 20/LO/0344. IRAS project ID 275470. Registered at the ISRCTN registry (ISRCTN34481181). Ethical approval is further detailed in the full EVIDENCE-PAH UK protocol (9).

## Results

### Baseline demographics

From the total EVIDENCE PAH population (*n* = 2,929), 1,991 individual records with recorded TRV values were identified for analysis (68.0%). The median time between echocardiography and right heart catheterisation was 40 days (Q1–Q3 6–106 days). Echo characteristics of non TRV dataset can be found in supplementary table 3. Characteristics of the population according to mPAP category (<21 mmHg, 21–24 mmHg and ≥25 mmHg) are displayed in Table 1. 1,414 individuals (71.0%) in the sample had confirmed pulmonary hypertension by ESC/ERS 2022 guidelines on haemodynamic testing, with 577 (29.0%) having mPAP < 21 mmHg, 472 (23.7%) having mPAP 21–24 mmHg, and 942 (47.3%) having mPAP ≥ 25 mmHg. Median age in each group was 63 years (51–72 years), 66 years (55–74 years), and 68 years (58–75 years), respectively. There was a female preponderance in all groups (71.4% of the mPAP < 21 mmHg group, 67.6% of the mPAP 21–24 mmHg group, and 65.3% of the mPAP ≥ 25 mmHg group).

**Table 1:** Baseline characteristics and echocardiographic metrics by mPAP group

Variable	mPAP (mmHg)			p- value
	<21 n = 577	21–24 n = 472	≥25 n = 942	
Age	63 (51–72)	66 (55–74)	68 (58–75)	<0.001
Female	412 (71.4%)	319 (67.6%)	615 (65.3%)	>0.05
mPAP (mmHg)	17 (15–19)	23 (22–24)	37 (30–47)	
TRV (ms^−1^)	2.67 (2.39–2.96)	2.95 (2.65–3.3)	3.54 (3.03–4.1)	<0.001
sPAP (mmHg)	32 (27–40)	40 (33–48)	59 (44–76)	<0.001
RAP (mmHg)	4.5 (3–6)	6.0 (4–8)	10.0 (7–14)	
	**<21** **n = 254**	**21–24** **n = 216**	**≥25** **n = 489**	
TAPSE (mm⋅mmHg^−1^)	21 (18–24)	21 (17.1–25)	17.3 (14–22)	<0.001
TAPSE/SPAP	0.65 (0.51–0.82)	0.51 (0.40–0.68)	0.29 (0.19–0.46)	<0.001

mPAP = mean pulmonary arterial pressure. RAP = right atrial pressure. SPAP = systolic pulmonary artery pressure. TAPSE = tricuspid annular plane systolic excursion. TRV = tricuspid regurgitation velocity

### Baseline echocardiographic metrics

**Fig. 1:**
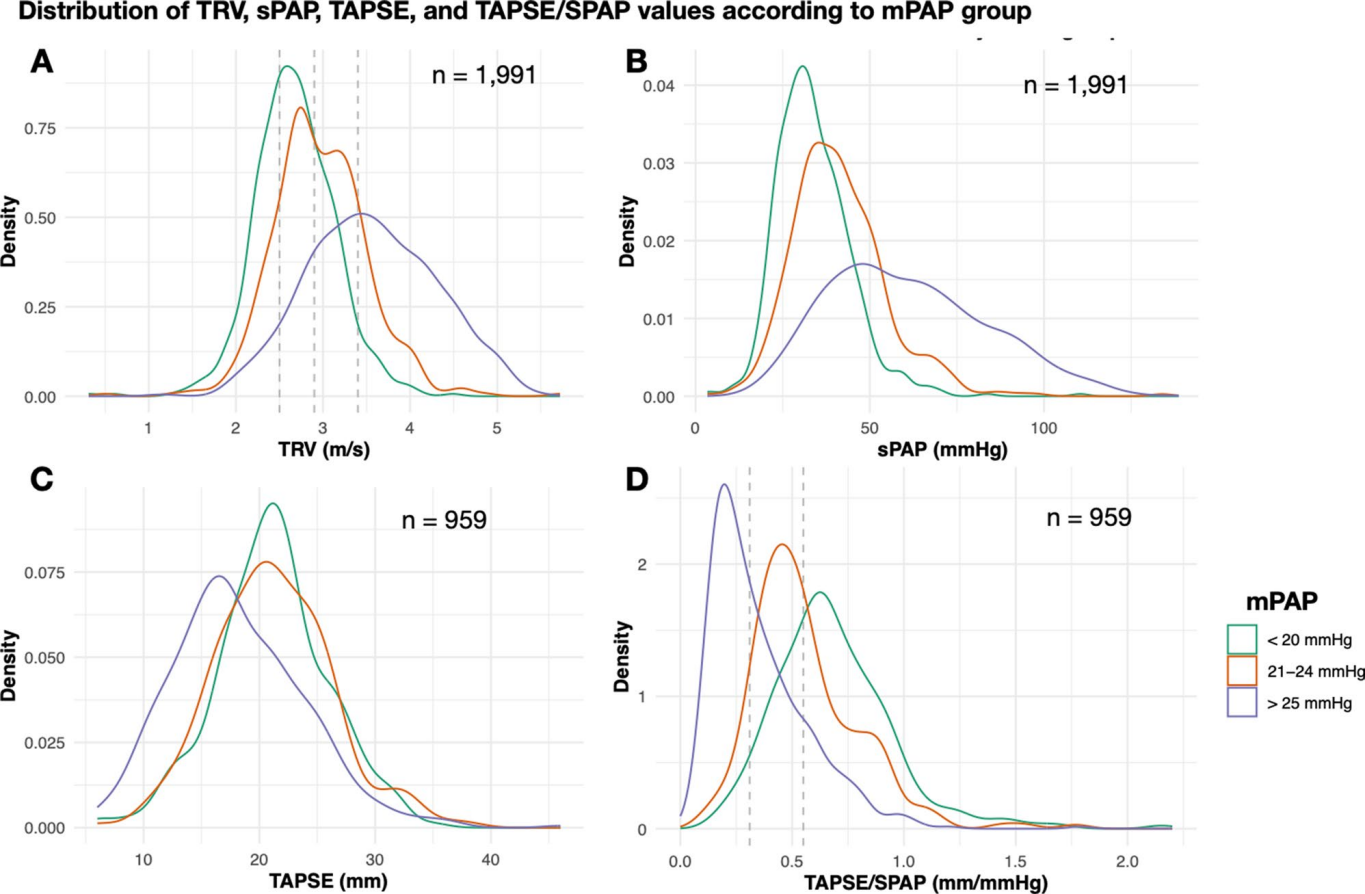
Histograms demonstrating distribution of (**A**) TRV, (**B**) TAPSE, and (**C**) TAPSE/SPAP values by mPAP group. mPAP = mean pulmonary artery pressure. SPAP = systolic pulmonary artery pressure. TAPSE = tricuspid annular plane systolic excursion. TRV = tricuspid regurgitation velocity

TRV, TAPSE, and TAPSE/SPAP distributions are displayed by mPAP group in Fig. 1.

TRV incremented with increasing haemodynamic group, being 2.67 m/s in the mPAP < 21 mmHg group, 2.95 m/s in the mPAP 21–24 mmHg group, and 3.54 m/s in the mPAP ≥ 25 mmHg group. Comparison of TRV values across groups showed a significant difference (*p* < 0.001) according to Fisher’s ANOVA. Further testing by Tukey multiple comparison test demonstrated significant differences in each group-wise comparison (*p* < 0.001 for all, see Supplementary Table 4).

Median right atrial pressure (RAP) incremented with each haemodynamic group, being 4.5 mmHg, 6.0 mmHg, and 10.0 mmHg, respectively (see Supplementary Table 5 for RAP groups by mPAP). Median sPAP also increased with each haemodynamic group, at 32 mmHg in the mPAP < 21 mmHg group, 40 mmHg in the 21–24 mmHg group and 59 mmHg in the ≥25 mmHg group (*p* < 0.001).

In the mPAP < 21 mmHg group, 254 (44.0%) individuals had TAPSE available, while in the mPAP 21–24 mmHg group and in the mPAP ≥ 25 mmHg group there were TAPSE values for 216 (45.8%) and 489 (51.9%) individuals, respectively. Median TAPSE for each haemodynamic group was 21 mm, 21 mm, and 17 mm, respectively, with TAPSE being reduced in only the mPAP ≥ 25 mmHg group. Comparison of TAPSE values across groups also showed a significant difference according to Fisher’s ANOVA (*p* < 0.001). However, Tukey’s test demonstrated no significant difference in TAPSE distribution between the mPAP < 21 mmHg and 21–24 mmHg groups (*p* = 0.974, compared to *p* < 0.001 for <21 vs ≥ 25 mmHg and 21–24 vs ≥ 25 mmHg, see Supplementary Table 6). This is demonstrated in the density plots, with considerable overlap between TAPSE in the mPAP < 21 mmHg and mPAP 21–24 mmHg group (see Fig. 1).

Results for TAPSE/SPAP are also displayed in Table 1. Median TAPSE/SPAP values were 0.65 mm/mmHg in the mPAP < 21 mmHg group, 0.51 mm/mmHg in the 21–24 mmHg group, and 0.29 mm/mmHg in the ≥25 mmHg group. Group-wise testing of TAPSE/SPAP between each group by Games–Howell test demonstrated significant differences between each group (*p* = 0.02 for mPAP < 21 vs 21–24 mmHg, *p* < 0.001 for <21 vs ≥ 25 mmHg, and *p* < 0.001 for 21–24 vs ≥ 25 mmHg, see Supplementary Table 1,7).

All other metrics can be found in Supplementary Table 8. Echocardiographic metrics by PH classification can be found in Supplementary Table 9. Both TRV and TAPSE/sPAP ratio show worse trends in the combined pre/post capillary PH, followed by pre-capillary PH compared to no PH and isolated post capillary PH (see Supplementary Table 8).

### TRV and TAPSE/SPAP as diagnostic values

Table 2 demonstrates the distribution of patients within haemodynamic groups by TRV and TAPSE/sPAP thresholds. The proportion of patients with low probability of PH by TRV (≤2.8 m/s) on echocardiography in the mPAP < 21 mmHg group was 69.5%, compared to 46.8% in the mPAP 21–24 mmHg group and 18.7% in the mPAP ≥ 25 mmHg group. The proportion of patients with intermediate probability of PH by TRV (2.9–3.4 m/s) was 25.1% in the mPAP < 21 mmHg group, 34.8% in the mPAP 21–24 mmHg group, and 24.5% in the mPAP ≥ 25 mmHg group. Lastly, the proportion of patients with high probability of PH by TRV (>3.4 m/s) was 5.4% in the mPAP < 21 mmHg group, 17.8% in the mPAP 21–24 mmHg group, and 56.7% in the mPAP ≥ 25 mmHg group. The proportions of patients in the intermediate to high probability of PH category (most likely to be put forward for invasive haemodynamics) were 30.5% in the mPAP < 21 mmHg, 52.6% in the mPAP 21–24 mmHg and 81.2% in the mPAP ≥ 25 mmHg groups.

**Table 2:** TRV and TAPSE/sPAP categories by mPAP group

(a) **Variable** TRV	mPAP (mmHg)			p- value
	<21 n = 577	21–24 n = 472	≥25 n = 942	
TRV < 2.8 ms^−1^	401 (69.5%)	221 (46.8%)	176 (18.7%)	*p* < 0.001
TRV 2.8–3.4 ms^−1^	145 (25.1%)	162 (34.8%)	228 (24.5%)	
TRV > 3.4 ms^−1^	31 (5.4%)	83 (17.8%)	528 (56.7%)	
(b) **Variable** **TAPSE/sPAP (mm/mmHg)**	**mPAP (mmHg)**			**p- value**
	**<21** **n = 254**	**21–24** **n = 216**	**≥25** **n = 489**	
Diagnostic (<0.55)	74 (29.1%)	125 (57.9%)	400 (81.8%)	<0.001
Prognostic (<0.31)	12 (4.72%)	17 (7.87%)	258 (52.76%)	<0.001

mPAP = mean pulmonary artery pressure. sPAP = systolic pulmonary artery pressure. TAPSE = tricuspid annular plane systolic excursion. TRV = tricuspid regurgitation velocity

The proportion with TAPSE/SPAP values who met the diagnostic threshold (0.55 mm/mmHg) in each group was 29.1%, 57.9%, and 81.8%, respectively.

**Fig. 2:**
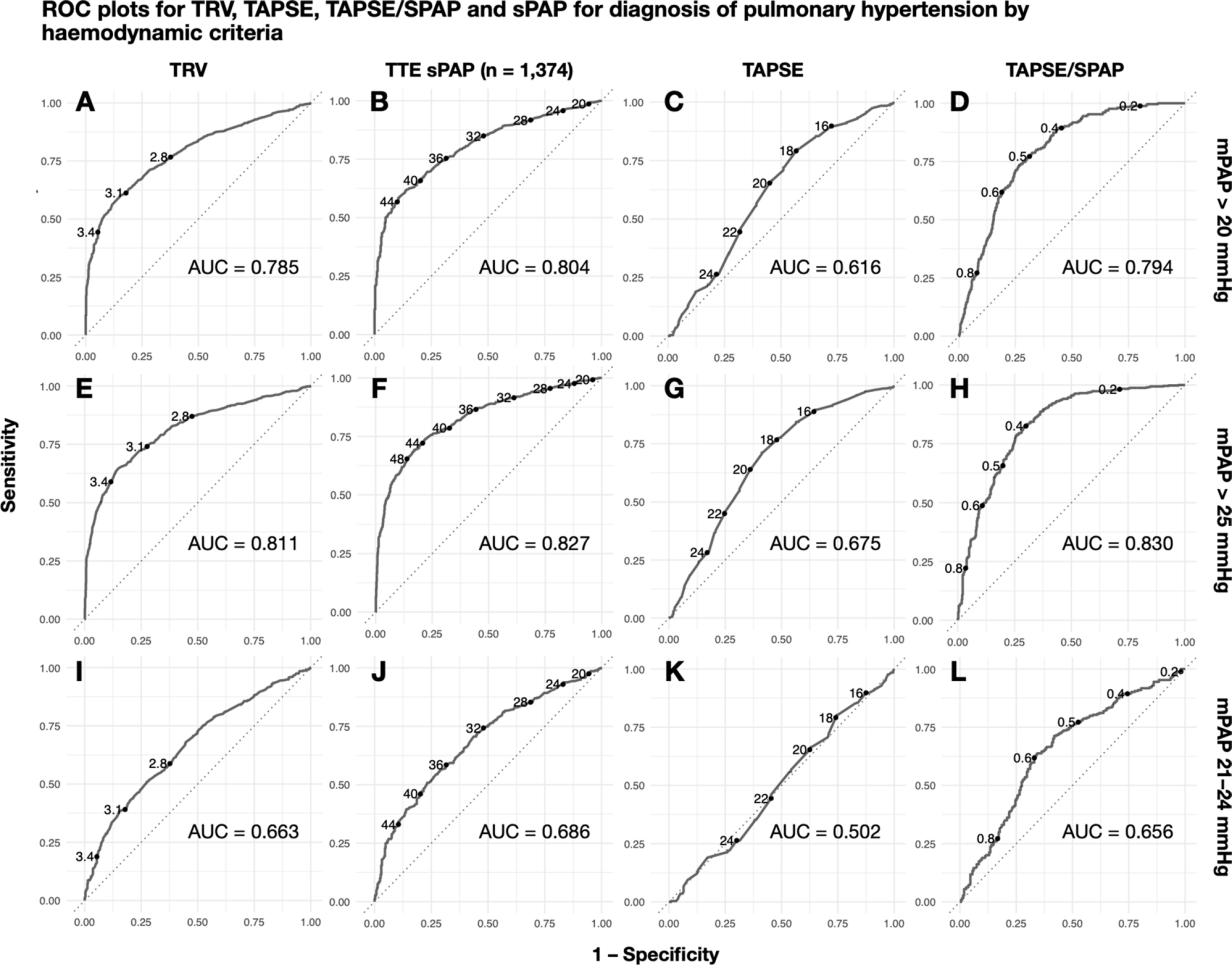
Receiver operating characteristic (ROC) plots for TAPSE/SPAP (**A**, **C**, **E**) and TRV (**B**, **D**, **F**) for diagnosis of pulmonary hypertension by haemodynamic criteria. AUC = area under curve. mPAP = mean pulmonary artery pressure. SPAP = systolic pulmonary artery pressure. TAPSE = tricuspid annular plane systolic excursion. TRV = tricuspid regurgitation velocity

In order to assess TRV, sPAP, TAPSE and TAPSE/SPAP ratio as diagnostic markers, ROC analysis was performed. TRV, sPAP, TAPSE and TAPSE/SPAP ratio were tested for prediction of mPAP > 20 mmHg, mPAP ≥ 25 mmHg, and mPAP 21–24 mmHg (see Fig. 2). TRV, sPAP and TAPSE/SPAP all performed well at predicting mPAP ≥ 25 mmHg, with area-under-curve (AUC) values of 0.811. 0.827 and 0.830, respectively (Fig. 2E, F and H). Sensitivity and specificity values when including patients with a mPAP > 20 mmHg were less, with AUC values of 0.785, 0.804 and 0.794 for TRV, sPAP and TASPE/sPAP, respectively (Fig. 2A, B and D). To test prediction of mPAP 21–24 mmHg, individuals with mPAP ≥ 25 mmHg were excluded. TRV, sPAP and TAPSE/sPAP performed less well at differentiating patients with a mPAP 21–24 mmHg from patients without PH, with AUC values of 0.663, 0.686 and 0.656, respectively (Fig. 2I, J and L). TAPSE/sPAP in patients with a TRV < 3.4 m/s (i.e. low and intermediate probability) did not add diagnostic value with AUC values of 0.677 in the mPAP > 20 mmHg group, 0.689 in the mPAP ≥ 25 mmHg group and 0.630 in the mPAP 21–24 mmHg group (Supplementary Figure 1).

Although TRV, sPAP and TAPSE/sPAP performed similarly across haemodynamic groups, DeLong’s test comparing TRV and sPAP, in mPAP > 20 mmHg and mPAP > 25 mmHg demonstrated superior performance by sPAP (*p* = 0.02). There was no significant difference between diagnostic methods in the mPAP 21–24 mmHg group (*p* = 0.3, see Supplementary Table 10).

TAPSE alone demonstrated the least sensitivity and specificity with AUC values, being 0.616 for mPAP > 20 mmHg, 0.675 for mPAP > 25 mmHg, with no utility in the mPAP 21–24 mmHg group with an AUC 0.502 (Fig. 2C, G and K). Reviewing TRV, sPAP, TAPSE, TAPSE/sPAP, no metric allowed differentiation between pre and post capillary PH as demonstrated into the supplementary ROC curves (Supplementary Figure 2).

### Survival analysis

Cox regression demonstrated significant effects of TRV, sPAP, TAPSE, and TAPSE/SPAP on survival when adjusted for individuals’ age and sex, with hazard ratios (HRs) of 1.646 (95% CI 1.514–1.788), 1.017 (95% CI 1.014–1.019), 0.941 (95% CI 0.925–0.958), and 0.192 (95% CI 0.126–0.292), respectively. A multivariate Cox regression model for survival was constructed with age, sex, TRV, and TAPSE as covariates. TRV and sPAP were found to have significant positive collinearity and so sPAP was excluded as a covariate, as it was the more derived method. TRV and TAPSE remained significant in multivariate analysis when adjusted for age and sex with HRs of 1.577 (95% CI 1.396–1.783) and 0.956 (95% CI 0.939–0.973), respectively (see Table 3).

**Table 3:** Cox regression analysis of survival for all individuals (*n* = 1,991)

Univariate* Cox regression models				Multivariate Cox regression model			
Variable	HR	95% CI	p	Variable	HR	95% CI	p
TRV	1.646	1.514–1.788	<0.001	Age	1.034	1.027–1.043	<0.001
sPAP	1.017	1.014–1.019	<0.001	Male sex	1.445	1.199–1.741	<0.001
TAPSE	0.941	0.925–0.958	<0.001	TRV	1.577	1.396–1.783	<0.001
TAPSE/SPAP	0.192	0.126–0.292	<0.001	TAPSE	0.956	0.939–0.973	<0.001

* Univariate analysis includes the named variable, adjusted for age and sex. CI = confidence interval. HR = hazard ratio. mPAP = mean pulmonary artery pressure. sPAP = systolic pulmonary artery pressure. TAPSE = tricuspid annular plane systolic excursion. TRV = tricuspid regurgitation velocity

**Fig. 3:**
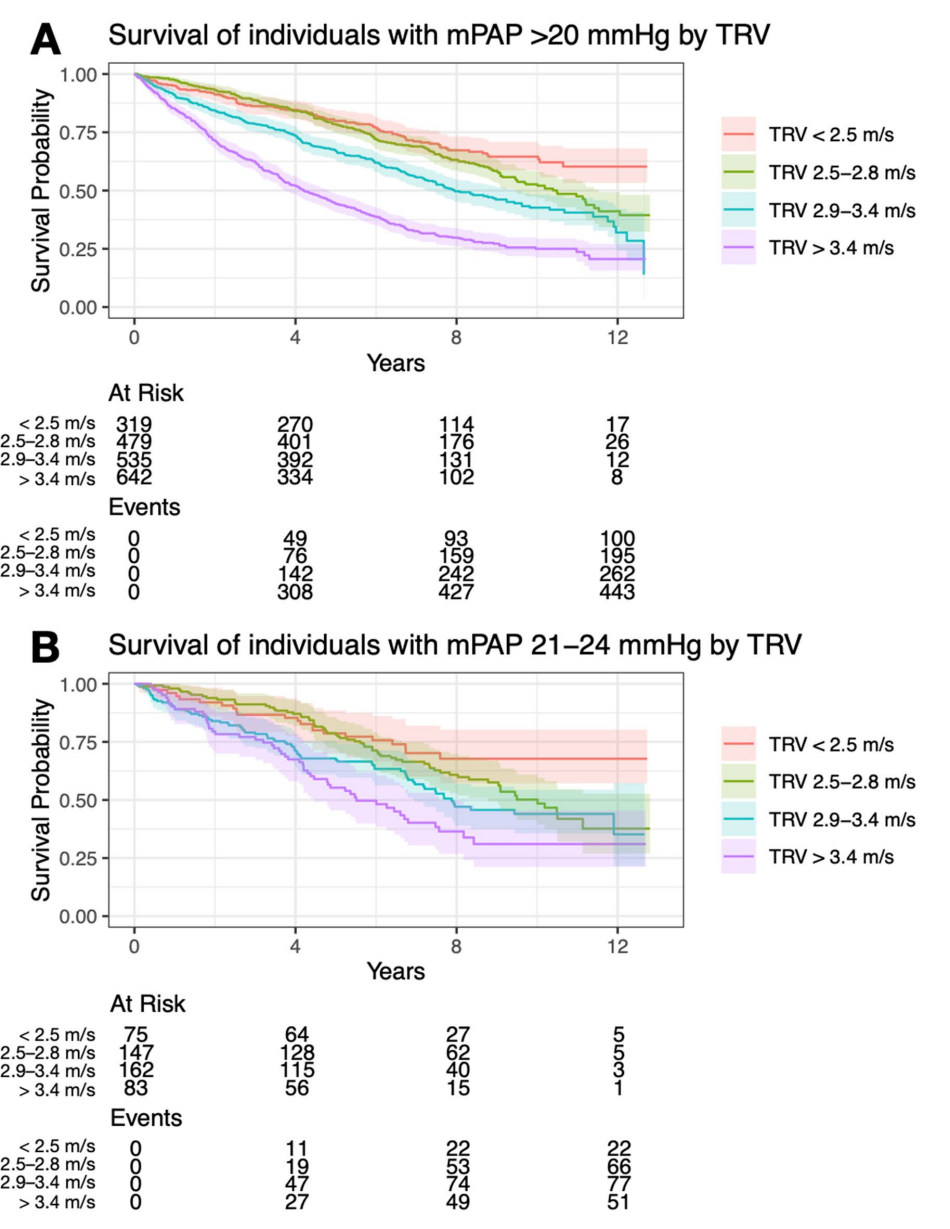
Kaplan–Meier graph for survival according to mPAP group across follow-up with risk table. Shaded areas represent 95% confidence intervals. MPAP = mean pulmonary artery pressure

Survival was also investigated according to TRV values in the mPAP 21–24 mmHg subgroup, with Kaplan-Meier survival curves shown in Fig. 3. Individuals were separated into groups with TRV values < 2.5 m/s, 2.5–2.8 m/s, 2.9–3.4 m/s, and >3.4 m/s. Median survival in the TRV 2.5–2.8 m/s group was 10.0 years, in the TRV 2.9–3.4 m/s group was 7.9 years, and in the TRV > 3.4 m/s group was 5.5 years. More than 50% of the TRV < 2.5 m/s group survived the follow-up period so median survival cannot be reported. One-year survival in each group was 96.0%, 98.0%, 90.1%, and 89.2%, respectively. Five-year survival in each group was 78.7%, 78.8%, 67.9%, and 55.3%, respectively. Pair-wise comparison revealed significant differences in survival between individuals with TRV < 2.5 and >3.4 m/s (*p* < 0.001), those with TRV 2.5–2.8 and >3.4 m/s (*p* < 0.001), and those with TRV < 2.5 and 2.9–3.4 m/s (*p* = 0.015) in the mPAP 21–24 mmHg group (Supplementary Table 11). In patients with PH as per the ESC/ERS 2022 guidelines (i.e. mPAP > 20 mmHg), pair-wise comparison revealed a difference across all TRV groups (*p* < 0.04, see Supplementary Table 12).

**Fig. 4:**
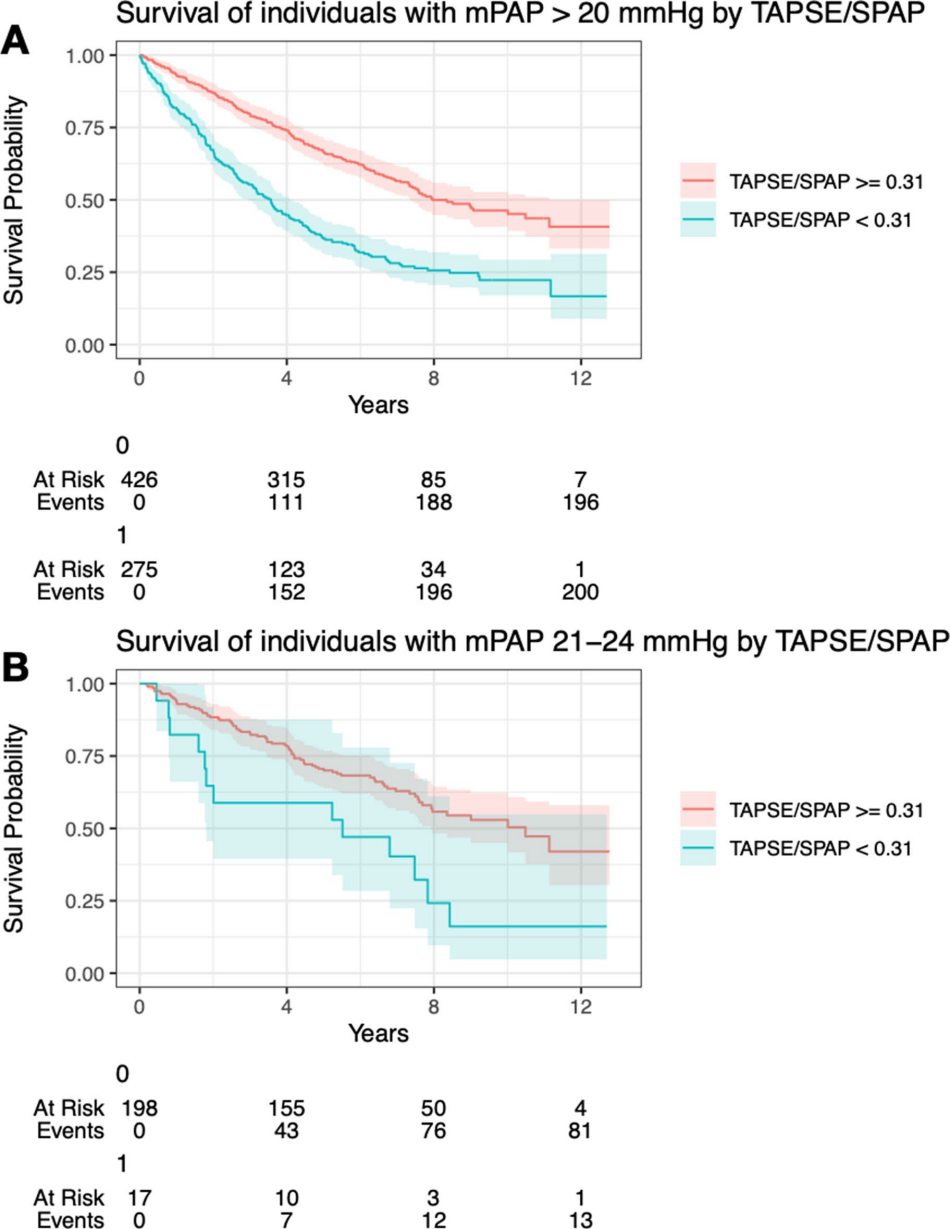
Kaplan–Meier graphs for survival according to TAPSE/SPAP group in (**A**) individuals with mPAP > 20 mmHg and (**B**) individuals with mPAP 21–24 mmHg. Risk tables are included. Shaded areas represent 95% confidence intervals. mPAP = mean pulmonary artery pressure. SPAP = systolic pulmonary artery pressure. TAPSE = tricuspid annular plane systolic excursion

Survival by TAPSE/SPAP threshold survival was investigated according to TAPSE/SPAP using a prognostic threshold of 0.31 mm/mmHg (Bello et al. 2019) in the mPAP 21–24 mmHg, with survival curves shown in Fig. 4B. In this group, median survival for individuals with TAPSE/SPAP ≥ 0.31 mm/mmHg was 10.5 years, while median survival for individuals with TAPSE/SPAP < 0.31 mm/mmHg was 5.5 years. There was a significant difference in survival between groups (*p* = 0.005 by log-rank test).

For all individuals with mPAP ≥ 20 mmHg, the proportion with TAPSE/SPAP values that met the prognostic threshold (0.31 mm/mmHg) in each group was 4.72%, 7.87%, and 52.8%, respectively. Survival curves for each group are displayed in Figure 4A. Median survival for individuals with TAPSE/SPAP ≥ 0.31 mm/mmHg was 8.0 years, while median survival for individuals with TAPSE/SPAP < 0.31 mm/mmHg was 3.6 years. There was a significant difference in survival between groups (*p* < 0.001 by log-rank test).

## Discussion

To our knowledge this study represents the largest cohort of patients with echo metrics within the newly defined lowered haemodynamic thresholds for PH (mPAP 21–24 mmHg) as defined by the ESC/ERS 2022 guidelines in a real-life referral population. Importantly, all patients had validation with invasive haemodynamic assessment alongside echocardiographic parameters.

Our data reveals progressive increase in TRV and sPAP with worsening haemodynamics, with the highest values observed in the population with mPAP ≥ 25 mmHg, consistent with previously published data. While there remains a significant difference between the population without pulmonary hypertension (mPAP < 21 mmHg) and the population with haemodynamically mild PH (mPAP 21–24 mmHg), density curves reveal significant overlap at these lower pressures. The sensitivity and specificity of TRV and sPAP drops in the mPAP 21–24 mmHg group compared to their performance across the entire population (i.e. mPAP > 20 mmHg) likely due to the presence individuals with more severe haemodynamics skewing the data.

TRV remains the cornerstone in the stratification and diagnosis of PH, endorsed by ESC/ERS and BSE guidelines [1, 4, 7]; our data supports this. Population studies have demonstrated TRV ≥ 2.9 m/s is the upper limit of normal [6, 18]. There are some data sets that report a lower threshold in healthy individuals [19]. However, one of the landmark echo studies in this area looking at tricuspid regurgitation gradient (TRG = 4(TRV^2^), a measure that correlates with invasive sPAP) did not support lowering of thresholds, revealing a lower positive predictive value using accepted TRG thresholds at lower haemodynamic thresholds [20]. While lowering thresholds may increase sensitivity, it leads higher false positive rates, and this risks clinical services being overwhelmed by patients who do not actually have PH. However, our data set also demonstrates that 46.8% of patients in the mPAP 21–24 mmHg group, would have been categorised as low probability of PH by TRV alone. In addition, there is evidence to suggest below traditional threshold [9] may still be prognostically significant. Our data set supports this with a mPAP of >20 mmHg, however, not in the mild elevations of mPAP group (mPAP 21–24 mmHg), where a threshold of >2.8 m/s demonstrates worsening survival compared to a TRV < 2.5 m/s.

Interestingly, sPAP performs better than TRV for predicting mPAP > 20 mmHg and >25 mmHg. This goes against studies that suggest inaccuracies related to RAP data [1, 11, 12]. One could speculate that reporting RAP may be more accurate as protocols have become more consistent and that combining two pressure-based metrics (TRG + RAP) may enhance diagnostic performance and skew towards identification of more severe haemodynamics.

Although indirect signs of PH remain important, as per other studies added value in mPAP > 20 mmHg is modest [6]. In our dataset, TAPSE only falls in patients with a mPAP > 25 mmHg, that is later in the disease process when right ventricular (RV) function becomes adversely affected.

Our study confirms findings previously reported in other major echocardiographic studies [6, 20] within the newly defined mild elevation haemodynamic group. Notably, D’Alto et al., in a study including 17 patients with mildly elevated haemodynamics and 23 with normal pulmonary pressures, demonstrated in a multivariable analysis that TRV ≥ 2.9 m/s was predictive of mPAP > 20 mmHg [6]. While reassuring, the dominant effect of the much larger population with an mPAP > 25 mmHg cannot be ignored in this kind of analysis. Both recent studies focusing on mPAP > 20 mmHg are dominated by the much larger population with an mPAP > 25 mmHg and as a result the data is skewed by patients with more severe haemodynamics [6, 20]. The EVIDENCE-PAH UK cohort provides sufficiently large sample size to focus specifically on patients with mild elevations in haemodynamics, mPAP 21–24 mmHg, allowing for a more granular evaluation of the diagnostic performance of both individual and combined echocardiographic parameters within this specific subgroup.

Our TAPSE/sPAP data demonstrates a higher proportion of patients meeting the diagnostic threshold the more severe the haemodynamic group. Observing 57.9% and 81.8% met the diagnostic threshold, in patients with a mPAP 21–24 mmHg and >25 mmHg, respectively, while 29.1% of patients in the no-PH group (mPAP < 21 mmHg) also met this criterion, giving a relatively high false positive rate. However, our ROC curves again reveal a reduction in both sensitivity and specificity of the diagnostic cut-off of 0.55 mm/mmHg when tested alone in the mPAP 21–24 mmHg group. Both sensitivity and specificity hold in the mPAP > 20 mmHg group, however this falls when one analysis the population with mild elevation in haemodynamics, mPAP 21–24 mmHg. Although intuition would suggest that TAPSE/sPAP, as a marker, of RV-PA coupling, may play a more significant role, it is likely the lack of change in TAPSE until more severe haemodynamics reduces its diagnostic utility in this mildly elevated haemodynamic group (mPAP 21–24 mmHg). Fewer patients in mild elevations of haemodynamics fall below the prognostic threshold < 0.31 mm/mmHg, however, the data still supports the prognostic threshold in the mild elevation group. There is evidence to suggest that TAPSE/sPAP improves risk stratification particularly in the intermediate risk group and this may translate to the mildly elevated haemodynamic group [21].

Most severe invasive and non-invasive measures of haemodynamics were observed in patients with combined pre-post capillary PH, a group that is often particularly challenging to manage, due to the high burden of comorbidity. Even amongst patients classified as no PH or isolated post-capillary PH based on haemodynamics, a significant proportion exhibit structural heart abnormality or right sided abnormality. This suggests that these individuals are not truly “normal”, instead representing a clinically unwell population, consistent with typical referral patterns to tertiary PH centres for right heart catheterisation, with unexplained symptoms, which meet the criteria for PH referral. This also highlights, that although echocardiographic metrics may differentiate between haemodynamic groups, this does not discriminate PH versus treatable PAH. Thus, lowering thresholds, like we see in EVIDENCE-PAH [17] may lead to increased referral rates of non-treatable PH with significant comorbidity identified.

The decision not to lower thresholds avoids increasing the false positive rates leading to unnecessary right heart catheterisation, this comes at the cost of failing to identify a significant proportion of patients with mildly abnormal haemodynamics, and thus diagnosis at earlier stages of the disease process.

To conclude, in milder forms of PH (mPAP 21–24 mmHg), we demonstrate that both single and combined metrics are not as good a predictor for diagnosis. This highlights the importance and intricacies of clinical and multimodality investigations and bringing together information in the diagnosis of PH, especially at lower haemodynamic thresholds. If there is clinical suspicion or Group 1 and 4 risk factors for PH, in the absence of echo metrics the gold standard of right heart catheterisation should still be considered. Importantly, our data demonstrates the prognostic utility of TRV and TAPSE/SPAP even in early haemodynamic disease.

### Limitation

EVIDENCE PAH UK was a haemodynamic database. Although a wealth of data, echo parameters were supplementary. Measurements were taken from reports and not raw data and re-analysis of echo images. As result although we focus on doppler measurements, additional supporting echo metrics are lacking in detail in sufficient numbers of patients to be analysed. As a result, there was missingness in the data set. Focused echo registries are important where newer multiparameter such as right ventricular free wall longitudinal strain (RVFWLS) and right ventricular fractional area of change (RVFAC) as reviewed IMPULSE [22] (IRAS Project ID: 323768) show great promise and maybe a more sensitive marker compared to TAPSE. RVFWLS/sPAP as a marker of RV coupling has also been found to be more powerful compared to other metrics [23]. The increased sensitivity may improve detection at mild elevations of haemodynamics, compared to conventional metrics.

Although we have excluded patients who do not have a TRV measurement, not having a TRV does not mean no PH. Even in the presence of PH around 15% have no measurable TRV [22]. Supplementary table 3, shows echo metrics where no TRV was available.

Guidelines have also changed over time. As a result, we are applying new guidelines to retrospective data, where referral patterns may have differed. We have also better standardised PH protocols. An example of this RAP, which used to be reported as a range, reporting has subsequently standardised. Chamber size, such as LA/RA, has changed over time from diameter, area and volumes. As a result in this data set which spans between 2009 and 2017, there are various ways of reporting same variables. Although there are methods to make this comparable, makes it difficult to compare more up to date measures/quantitative measures.

A key limitation of our study is that echocardiography and right heart catheterisation were not performed simultaneously. Echocardiographic assessments were included if conducted within one year prior to, or within three months following, catheterisation. Although we ensured no new targeted therapies had been initiated during this interval, this temporal separation may have introduced variability in haemodynamic and echocardiographic measurements.

By default, patients will have been deemed high enough risk of PH to undergo catheterisation even where the echo parameters were reassuring. Therefor we are slightly biased towards a higher clinical suspicion of PH in this study.

## Electronic supplementary material

Below is the link to the electronic supplementary material.

Supplementary Material 1

Supplementary Material 2

## Data Availability

To allow independent interpretation of the clinical study results, all clinical authors had access to anonymised data, to fulfil their roles under the ICMJE criteria. To allow for full transparency, researchers can request access to study data after publication.
