# Supplementary material for: Significance of echocardiographic metrics including TRV and TAPSE/SPAP in mild haemodynamic pulmonary hypertension – data from EVIDENCE-PAH UK
Source: Echo Res Pract. 2026 Jun 8;13:20. doi: 10.1186/s44156-026-00119-1 (PMC13245079; doi:10.1186/s44156-026-00119-1)
Supplement: Supplementary file 1 — Supplementary Material 1 [file 44156_2026_119_MOESM1_ESM.docx]

**Supplementary Figure 1**





Receiver operating characteristic (ROC) curves for TAPSE/SPAP and SPAP calculated using TTE-estimated RAP as a predictor of mPAP category in the entire cohort versus individuals with TRV ≤ 3.4 m/s. Note that as lower TAPSE/SPAP values are hypothesised to predict higher mPAP, the ROC curves actually represent prediction of the non-disease state. AUCs remain the same as for predicting the disease state.

**Supplementary Figure 2**


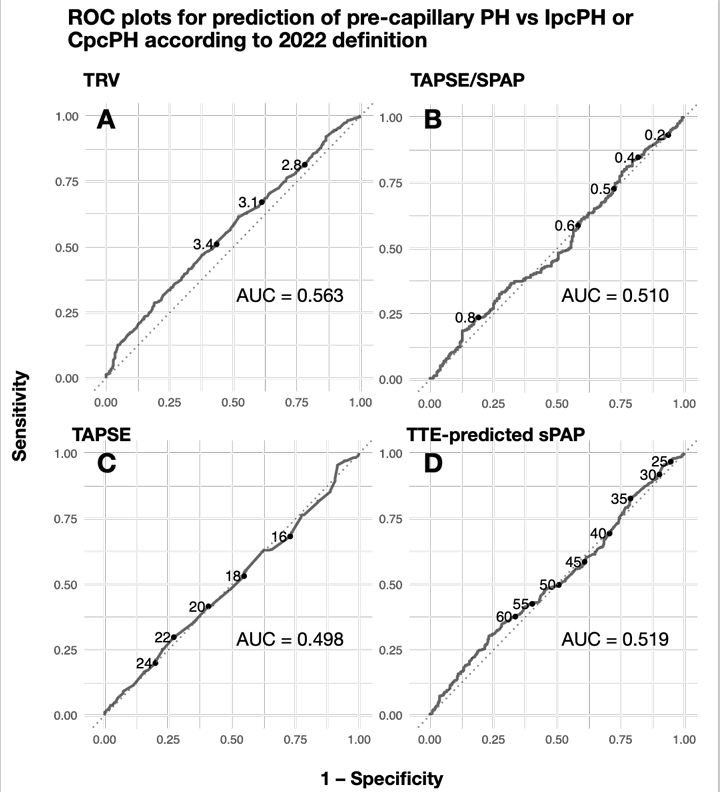


Receiver operating characteristic (ROC) curves for TRV, TAPSE/SPAP, TAPSE, and SPAP (as calculated using TTE-predicted RAP) for the prediction of pre-capillary PH vs combined IpcPH or CpcPH.
