# Supplementary material for: Significance of echocardiographic metrics including TRV and TAPSE/SPAP in mild haemodynamic pulmonary hypertension – data from EVIDENCE-PAH UK
Source: Echo Res Pract. 2026 Jun 8;13:20. doi: 10.1186/s44156-026-00119-1 (PMC13245079; doi:10.1186/s44156-026-00119-1)
Supplement: Supplementary file 2 — Supplementary Material 2 [file 44156_2026_119_MOESM2_ESM.docx]

**Supplementary Table 1**

| Variable | Result (mean + SD or median and Q1–Q3) | n | Missing |
| --- | --- | --- | --- |
| Age | 63.29 (14.05) | 1991 | 0 |
| mPAP | 24 (20–36) | 1991 | 0 |
| TRV | 3.07 (2.65–3.60) | 1991 | 0 |
| RA dilated | 1,165 (58.5%) | 1991 | 0 |
| RV dilated | 761 (39.5%) | 1928 | 63 |
| LA dilated | 548 (28.6%) | 1917 | 74 |
| LVH | 262 (13.8%) | 1904 | 87 |
| Low LVEF | 69 (3.55%) | 1943 | 48 |
| LV diastolic dysfunction | 1,169 (58.7%) | 1991 | 0 |
| MR present | 147 (8.24%) | 1783 | 208 |
| TAPSE | 20 (16–23) | 959 | 1032 |
| sPAP calculated from TTE estimated RAP | 41 (32–59) | 1374 (69%) | 617 |
| sPAP calculated from RHC measured RAP | 43 (33–60) | 617 (31%) | N/A |

**Supplementary Table 2**

| **Priority of qualitative and quantitative metrics used** | | | |
| --- | --- | --- | --- |
| Parameter | Priority | Metric | n |
| RA | 1 | RA Area | 522 |
|  | 2 | RA Diameter | 10 |
|  | 3 | RA Visual | 1459 |
| RV | 1 | RVD1 | 708 |
|  | 2 | RV Visual | 1215 |
|  | 3 | RV Missing | 68 |
| LA | 1 | LA Volume | 193 |
|  | 2 | LA Area | 132 |
|  | 3 | LA Diameter | 595 |
|  | 4 | LA Visual | 997 |
| LV diastolic | 1 | E/A or E/E’ | 449/314 |
|  | 2 | Diastolic grade | 192 |

**Supplementary Table 3**

**Number with noTRV values in EVIDENCE-PAH cohort**

| mPAP Group | ≤ 20 | 21–24 | ≥ 25 |
| --- | --- | --- | --- |
| Total | 968 | 689 | 1272 |
| TRV present | 577 (59.6%) | 472 (68.5%) | 942 (74.1%) |
| TRV not present | 391 (40.4%) | 217 (31.5%) | 330 (26.0%) |

|  | mPAP<20mmHg  n=391 | mPAP 21-24mmHg  n=217 | mPAP≥ 25  n=330 |
| --- | --- | --- | --- |
| TR present  No regurgitation  Mild regurgitation  Mild + regurgitation  Present unable to quantify  Missing | 215 (55.0%)  45 (11.5%)  32 (8.2%)  4 (1.0%)  95 (24.3%) | 110 (50.7%)  26 (12.0%)  22 (10.1%)  0 (0%)  59 (27.2%) | 103 (31.2%)  32 (9.7%)  83 (25.2%)  1 (0.3%)  111 (33.6%) |
| RAP (median)  Missing | 3.0  243 (62.1%) | 3.0  134 (61.8%) | 3.0  193 (58.5%) |
| RA size  Normal  Dilated  Missing | 221 (56.5%)  60 (15.3%)  110 (28.1%) | 118 (54.4%)  40 (18.4%)  59 (27.2%) | 89 (27.0%)  206 (62.4%)  117 (35.5%) |
| RV size  Normal  Dilated  Missing | 227 (58.1%)  70 (17.9%)  94 (24.0%) | 122 (56.2%)  41 (18.9%)  54 (24.9%) | 84 (25.5%)  137 (41.5%)  109 (33.0%) |
| TAPSE (median) | 22.0 | 21.8 | 19.0 |
| RV function visual  Normal  Impaired  Missing | 281 (71.9%)  23 (5.9%)  87 (22.3%) | 149 (68.7%)  17 (7.8%)  51 (23.5%) | 119 (36.1%)  102 (30.9%)  109 (33.0%) |
| LA size  Normal  Dilated  Missing | 200 (51.2%)  98 (25.1%)  93 (23.8%) | 92 (42.4%)  74 (34.1%)  51 (23.5%) | 118 (35.8%)  101 (30.6%)  111 (33.6%) |

**Supplementary Table 4**

| **Tukey multiple comparison test for TRV by mPAP group** | | | | |
| --- | --- | --- | --- | --- |
| Groups compared | diff | lwr | upr | p adj |
| ≤ 20 vs 20–24 | 0.291 | 0.201 | 0.381 | < 0.001 |
| ≤ 20 vs ≥ 25 | 0.886 | 0.809 | 0.962 | < 0.001 |
| 21–24 vs ≥ 25 | 0.594 | 0.512 | 0.676 | < 0.001 |

**Supplementary Table 5**

| **Variable** | | **Number in each  category by mPAP (mmHg)** | | |
| --- | --- | --- | --- | --- |
|  |  | **≤ 20** | **21–24** | **≥ 25** |
| RAP estimated by TTE (mmHg) | 3 | 326 (56.5%) | 275 (58.3%) | 354 (37.6%) |
|  | 8 | 27 (4.7%) | 49 (10.4%) | 132 (14.0%) |
|  | 15 | 19 (3.3%) | 29 (6.1%) | 163 (17.3%) |
|  | Missing | 205 (35.5%) | 119 (25.2%) | 293 (31.1%) |
| IVC size | Normal | 348 (60.3%) | 289 (61.2%) | 413 (43.8%) |
|  | Dilated | 42 (7.3%) | 66 (14%) | 260 (27.6%) |
|  | Missing | 187 (32.4%) | 117 (24.8%) | 269 (28.6%) |
| IVC response | Normal | 345 (59.8%) | 312 (66.1%) | 443 (47.0%) |
|  | Not collapsable | 30 (5.2%) | 41 (8.7%) | 210 (22.3%) |
|  | Missing | 202 (35%) | 119 (25.2%) | 289 (30.7%) |

**Supplementary Table 6**

| **Tukey multiple comparison test for TAPSE by mPAP group** | | | | |
| --- | --- | --- | --- | --- |
| Groups compared | diff | lwr | upr | p adj |
| ≤ 20 vs 20–24 | 0.109 | -1.064 | 1.283 | 0.974 |
| ≤ 20 vs ≥ 25 | -2.896 | -3.877 | -1.915 | < 0.001 |
| 21–24 vs ≥ 25 | -3.005 | -4.041 | -1.969 | < 0.001 |

**Supplementary Table 7**

| **Games–Howell multiple comparison test for TAPSE/SPAP by mPAP group** | | | | |
| --- | --- | --- | --- | --- |
| Groups compared | estimate | lwr | upr | p adj |
| ≤ 20 vs 20–24 | -0.113 | -0.211 | -0.015 | 0.020 |
| ≤ 20 vs ≥ 25 | -0.350 | -0.411 | -0.290 | < 0.001 |
| 21–24 vs ≥ 25 | -0.238 | -0.322 | -0.154 | < 0.001 |

**Supplementary Table 8**

| **Variable** | **mPAP (mmHg)** | | | **p-value** |
| --- | --- | --- | --- | --- |
|  | **≤ 20**  **n = 577** | **21–24**  **n = 472** | **≥ 25**  **n = 942** |  |
| RA Dilated | 193 (35.5%) | 195 (45.7%) | 631 (72.2%) | < 0.001 |
| RV Dilated | 124 (22.6%) | 137 (29.6%) | 500 (54.6%) | < 0.001 |
| LA Dilated | 129 (23.1%) | 115 (25.2%) | 304 (33.7%) | < 0.001 |
| LVH | 55 (10.1%) | 50 (10.9%) | 157 (17.4%) | 0.001 |
| Low LVEF | 17 (2.9%) | 15 (3.2%) | 37 (3.9%) | > 0.05 |
| LV Diastolic Dysfunction | 394 (68.3%) | 305 (64.6%) | 470 (49.9%) | < 0.001 |
| MR | 188 (37.5%) | 187 (43.3%) | 417 (49.1%) | < 0.001 |

Additional baseline characteristics and echocardiographic metrics by mPAP group. LA = left atrium. LV = left ventricle. LVEF = left ventricular ejection fraction. LVH = left ventricular hypertrophy. mPAP = mean pulmonary arterial pressure. MR = mitral regurgitation. RA = right atrium. RV = right ventricle.

**Supplementary Table 9**

| **2022 Definition** | | | | | | |
| --- | --- | --- | --- | --- | --- | --- |
|  | Pre-cap PH | IpcPH | CpcPH | Unclassified | Not PH | p |
| n | 791 (39.7%) | 186 (9.34%) | 270 (13.6%) | 168 (8.44%) | 576 (28.9%) |  |
| Age | 64.12 (13.33) | 63.2 (13.47) | 69.69 (12.28) | 59.21 (14.54) | 60.38 (14.68) | < 0.001 |
| mPAP | 30 (24–45) | 28 (24–32) | 39 (32–47.75) | 22 (21–24) | 17 (15–19) | < 0.001 |
| TRV | 3.4 (2.94–4.02) | 2.97 (2.63–3.32) | 3.57 (3.09–4.04) | 2.8 (2.55–3.24) | 2.67 (2.4–2.96) | < 0.001 |
| RA dilated | 512 (64.7%) | 117 (62.9%) | 230 (85.2%) | 80 (47.6%) | 226 (39.2%) | < 0.001 |
| RV dilated | 375 (48.4%) | 64 (36%) | 155 (59.8%) | 43 (25.6%) | 124 (22.6%) | < 0.001 |
| LA dilated | 188 (25%) | 58 (32.4%) | 123 (47.1%) | 50 (29.8%) | 129 (23.1%) | < 0.001 |
| LVH | 84 (11.1%) | 30 (17%) | 75 (28.6%) | 19 (11.4%) | 54 (10%) | < 0.001 |
| Low LVEF | 20 (2.5%) | 7 (3.8%) | 20 (7.4%) | 5 (3.0%) | 17 (3.0%) | 0.148 |
| LVDD | 441 (55.8%) | 99 (53.2%) | 107 (39.6%) | 129 (76.8%) | 393 (68.2%) | < 0.001 |
| MR | 36 (4.55%) | 20 (10.8%) | 44 (16.3%) | 13 (7.74%) | 34 (5.90%) | < 0.001 |
| n for TAPSE/SPAP | 414 | 94 | 126 | 67 | 254 |  |
| TAPSE | 18 (15–23) | 20 (18–25) | 16 (14–20) | 23 (20–25) | 21 (18–24) | < 0.001 |
| TAPSE/SPAP | 0.36 (0.21–0.52) | 0.47 (0.35–0.68) | 0.26 (0.18–0.36) | 0.57 (0.45–0.74) | 0.65 (0.51–0.82) | < 0.001 |
| **2015 Definition** | | | | | | |
|  | Pre-cap PH | IpcPH | CpcPH |  | Not PH | p |
| n | 551 (27.7%) | 245 (12.3%) | 146 (7.33%) |  | 1049 (52.7%) |  |
| Age | 63.18 (13.65) | 65.81 (12.06) | 71.08 (12.54) |  | 61.68 (14.45) | <0.001 |
| mPAP | 39 (29–50) | 32 (29–37) | 44 (38–53) |  | 20 (17–22) | < 0.001 |
| TRV | 3.68 (3.13–4.2) | 3.16 (2.81–3.5) | 3.88 (3.42–4.25) |  | 2.75 (2.5–3.14) | < 0.001 |
| RA dilated | 387 (70.2%) | 179 (73.1%) | 133 (91.1%) |  | 466 (44.4%) | < 0.001 |
| RV dilated | 300 (55.5%) | 109 (46.4%) | 91 (65%) |  | 261 (25.8%) | < 0.001 |
| LA dilated | 138 (26.4%) | 102 (42.9%) | 64 (45.4%) |  | 244 (24%) | < 0.001 |
| LVH | 65 (12.3%) | 48 (20.5%) | 44 (31.2%) |  | 105 (10.5%) | < 0.001 |
| Low LVEF | 13 (2.4%) | 12 (4.9%) | 12 (8.2%) |  | 32 (3.1%) | 0.007 |
| LVDD | 302 (54.8%) | 121 (49.4%) | 47 (32.2%) |  | 699 (66.6%) | < 0.001 |
| MR | 30 (5.44%) | 24 (9.80%) | 30 (20.5%) |  | 63 (6.01%) | < 0.001 |
| n for TAPSE/SPAP | 303 | 103 | 80 |  | 469 |  |
| TAPSE | 17 (14–22) | 20 (17–24) | 15 (13–17) |  | 21 (18–24) | < 0.001 |
| TAPSE/SPAP | 0.28 (0.18–0.45) | 0.43 (0.31–0.59) | 0.21 (0.15–0.28) |  | 0.59 (0.44–0.78) | < 0.001 |

Baseline characteristics and echocardiography values of our sample according to 2015/2022 ESC/ERS definitions. LA = left atrium. LV = left ventricle. LVEF = left ventricular ejection fraction. LVH = left ventricular hypertrophy. mPAP = mean pulmonary arterial pressure. MR = mitral regurgitation. RA = right atrium. RV = right ventricle. RAP = right atrial pressure. SPAP = systolic pulmonary artery pressure. TAPSE = tricuspid annular plane systolic excursion. TRV = tricuspid regurgitation velocity.

**Supplementary Table 10**

| **DeLong's Tests for TRV vs TTE sPAP ROC Curves** | | | | |
| --- | --- | --- | --- | --- |
|  | TRV AUC | sPAP AUC | Z-score | p |
| mPAP >20 | 0.795 | 0.804 | -2.290 | 0.02204 |
| mPAP >25 | 0.816 | 0.827 | -2.996 | 0.002735 |
| mPAP 21–24 | 0.680 | 0.686 | -1.009 | 0.3132 |
| According to Bonferroni's correction, alpha = 0.0167 | | | | |

**Supplementary Table 11**

| Group-wise survival by TRV of those with mPAP 21–24 mmHg (values are p-values) | | | |
| --- | --- | --- | --- |
| TRV (m/s) | < 2.5 | 2.5–2.8 | 2.9–3.4 |
| 2.5–2.8 | 0.10923 | – | – |
| 2.9–3.4 | 0.01541 | 0.10923 | – |
| > 3.4 | 0.00034 | 0.00065 | 0.07129 |
| Comparison by log-rank test with BH correction for multiple comparisons (i.e. alpha = 0.05) | | | |

**Supplementary Table 12**

| Group-wise survival by TRV of those with mPAP >20 mmHg (values are p-values) | | | |
| --- | --- | --- | --- |
| TRV (m/s) | < 2.5 | 2.5–2.8 | 2.9–3.4 |
| 2.5–2.8 | 0.0360 | – | – |
| 2.9–3.4 | 0.0000 | 0.0001 | – |
| > 3.4 | 0.0000 | 0.0000 | 0.0000 |
| Comparison by log-rank test with BH correction for multiple comparisons (i.e. alpha = 0.05) | | | |
